# The Impact of Air Pollution on Frequent Exacerbations among COPD Patients: An Observational Study on the Population of Western Romania

**DOI:** 10.3390/jcm11154352

**Published:** 2022-07-27

**Authors:** Gabriel-Petrică Bălă, Bogdan Timar, Florin Gorun, Radu Motisan, Camelia Pescaru, Emanuela Tudorache, Monica Marc, Diana Manolescu, Cosmin Citu, Cristian Oancea

**Affiliations:** 1 Center for Research and Innovation in Precision Medicine of Respiratory Diseases, “Victor Babes” University of Medicine and Pharmacy Timisoara, Eftimie Murgu Square 2, 300041 Timisoara, Romania; balagabriel1991@yahoo.ro (G.-P.B.); pescaru.camelia@umft.ro (C.P.); tudorache_emanuela@yahoo.com (E.T.); stel.marc@gmail.com (M.M.); oancea@umft.ro (C.O.); 2 Department of Internal Medicine II, Division of Diabetes, Nutrition and Metabolic Diseases, “Victor Babes” University of Medicine and Pharmacy Timisoara, Eftimie Murgu Square 2, 300041 Timisoara, Romania; 3 Center for Molecular Research in Nephrology and Vascular Disease, Faculty of Medicine, “Victor Babes” University of Medicine and Pharmacy Timisoara, Eftimie Murgu Square 2, 300041 Timisoara, Romania; 4 Department of Obstetrics and Gynecology, Municipal Emergency Clinical Hospital Timisoara, 1-3 Alexandru Odobescu Street, 300202 Timisoara, Romania; gorun.florin@umft.ro; 5 MagnaSCI SRL, 7 Luceafarul Street, 300414 Timisoara, Romania; radu.motisan@magnasci.com; 6 Department of Radiology, “Victor Babes” University of Medicine and Pharmacy Timisoara, Eftimie Murgu Square 2, 300041 Timisoara, Romania; dmanolescu@umft.ro; 7 Department of Obstetrics and Gynecology, “Victor Babes” University of Medicine and Pharmacy Timisoara, 300041 Timisoara, Romania; citu.ioan@umft.ro

**Keywords:** particulate matter, chronic obstructive pulmonary disease, air pollution, exacerbation

## Abstract

Patients with respiratory pathologies are the risk group most affected by air pollution, being directly exposed, especially those diagnosed with chronic obstructive pulmonary disease (COPD). In this observational study, which included 79 patients, we evaluated whether COPD patients with the frequent exacerbating phenotype or the infrequent exacerbating phenotype live in residences with higher values of air pollution. An air quality monitoring station was installed in each patient’s house for at least 24 h and PM 1.0, PM 2.5, and PM 10 were measured. Average PM 1.0, PM 2.5, and PM 10 values were lower in the group of infrequently exacerbating patients compared to the frequently exacerbating ones. For every 1 µg/m^3^ increase in the average values of PM 1.0, PM 2.5, and PM 10, there is an increase of 1.7%, 1.8% and 1%, respectively, in the risk of developing exacerbations. More importantly, an average value of PM 1.0, PM 2.5, and PM 10 above 32.21 µg/m^3^, 82.32 µg/m^3^ and 42.89 µg/m^3^ increases the probability of developing an exacerbation by 3.83, 10.14, and 4.12 times, respectively. Our analysis showed that COPD patients with a frequently exacerbating phenotype live in residences with high levels of air pollution compared to infrequently exacerbating ones.

## 1. Introduction

Chronic obstructive pulmonary disease (COPD) is the fourth leading cause of mortality worldwide and places a large burden on health systems [1,2]. Smoking is the most important risk factor for COPD, although air pollution also has a significant role in lung function impairment [1,2]. The disease is characterized by several phenotypes, including: emphysematous phenotype; chronic bronchial phenotype; asthma superimposed with COPD; bronchiectasis superimposed with COPD; and frequent exacerbation phenotype versus infrequent exacerbation phenotype [3,4]. Patients with the frequently exacerbating phenotype are characterized by the presence of at least two moderate exacerbations that require antibiotic therapy or systemic corticosteroid therapy, or at least one exacerbation that requires hospitalization, per year [3,4]. Acute exacerbation (AE) is a major cause of global death in patients with COPD and it can have infectious or non-infectious etiology. In a study conducted by Che et al., it was observed that an increased concentration of particulate matter may cause an increase in the degree and diversity of bacteria in bronchoalveolar lavage, thus potentiating an acute exacerbation [5].

The composition of air is diversified, and air pollutants can cause negative effects on the respiratory system. Ambient particulate matter (PM) has a complex composition, being divided due to its size: PM 2.5 represents particles with dimensions smaller than 2.5 µm, being more present in gaseous substances, and which are more susceptible to cause respiratory symptoms, while PM 10 has dimensions greater than 10 µm, and thus is found more in dust and has a probability of remaining in the upper respiratory tract [6,7]. In a meta-analysis conducted by Li et al., it was observed that for a 10 µg/m^3^ increase in PM 2.5, the number of hospitalizations of COPD patients increased by 3.1% [8]. Patients exposed to high concentrations of PM 2.5 were associated with the development of COPD, and their short-term exposure to high concentrations was corroborated with an increase in the number of exacerbations and hospitalizations [9]. At the same time, PM 1.0 is not sufficiently studied in the literature. The size of the particles is inversely proportional to the damage they cause to the lungs. The smaller their size, the more harmful they can be to the respiratory system and especially to the lungs. In the United States, there have been several requests for PM 1.0 to be considered a standard environmental risk factor. There were several concerns about whether this is a different risk factor compared to PM 2.5 and whether it could provide additional information on its role and health impairment. Several studies have shown that the origin of PM 1.0 is the same as that of PM 2.5 [9].

The aim of this study was to assess whether frequently exacerbating patients compared to infrequently exacerbating patients live in residences with higher values of air pollution. The reason we chose this comparison is that these patients have a faster decrease in lung function with each exacerbation, generating a very high consumption of financial resources and an increased risk of developing subsequent depression [4].

## 2. Materials and Methods

### 2.1. Study Design and Settings

An observational cohort study was conducted on patients diagnosed with COPD admitted to the Pulmonology Clinic of “Victor Babes” Clinical Hospital in Timisoara, Romania, to assess any correlation between microparticulate air pollution, atmospheric factors and COPD exacerbations. The study was conducted between September 2020 and March 2021.

The study was conducted according to the guidelines of the Declaration of Helsinki and approved by the Institutional Review Board of the Hospital of Infectious Diseases and Pneumophtisiology “Dr. Victor Babes”, Timisoara (No.6111/18.08.2020). Informed consent was obtained from all subjects involved in the study. Subsequently, home visits were organized for data collection, as well as the installation of air pollution monitoring equipment.

### 2.2. Participants

For this study, patients diagnosed with severe or very severe COPD (stages III and IV) according to the Global Initiative for Chronic Obstructive Pulmonary Disease (GOLD) (FEV1 < 50%) were included. These patients spent most of their time indoors, in the same room, constantly breathing the same air. Thus, the inclusion criteria were the following: (1) age over 45 years; (2) patients diagnosed for at least 1 year with COPD; (3) patients without acute exacerbations during monitoring; (4) severe to very severe COPD (FEV1 < 50%). Patients under 45 years of age, patients who had acute exacerbation, and patients with FEV1 values ≥ 50% or FEV1/FVC values ≥ 0.7 were excluded. The medication that patients received during the study was administered according to GOLD guidelines. During the study, patients were stable with no symptoms present.

### 2.3. Variables

Data were extracted by two researchers from patients’ electronic medical records using a standardized data collection form. Demographic elements and clinical data collected were gender, age, place of residence, smoking status, comorbidities, and spirometry data. The primary outcome of interest was the difference in air pollution level.

### 2.4. Data Sources/Measurement

**Figure 1:**
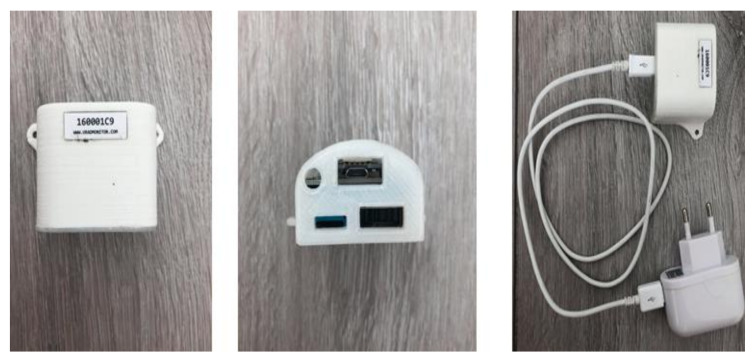
uRADMonitor SMOGGIE-PM.

Measurements of atmospheric parameters were obtained using a uRADMonitor SMOGGIE-PM (Magnasci SRL, Timisoara, Romania) (Figure 1).

The uRADMonitor SMOGGIE-PM is a device measuring 42 × 43 × 27 mm that can monitor air quality. This device has a water-resistant plastic frame and can be easily installed in houses, both indoors and outdoors. It has several high-precision laser sensors that can record the values of PM 10, PM 2.5, and PM 1.0, and additionally has sensors for humidity, atmospheric pressure and temperature. It connects to a power source using a standard 5V micro-USB power cord. The device connects to the internet through a WiFi connection and can transmit data in real time, which can be viewed centrally by accessing the uRADMonitor API or in a decentralized manner by using the local network.

The uRADMonitor SMOGGIE-PM measures the parameters every 60 s, being able to monitor them in real time. The device calculated the MIN, MAX and MEAN values during the recording period.

### 2.5. Statistical Analysis

Statistical analysis was performed using RStudio. Categorical variables were reported as absolute number (*n*) and observed frequency (%) and compared using Fisher’s exact test or linear-by-linear association. The Shapiro–Wilk test was used to evaluate the normality of the distribution values. Depending on the normality of the distribution, continuous variables were represented as a median (interquartile range) or as a mean (±SD). Mood’s median test was used to compare non-normally distributed variables while continuous variables with a normal distribution were compared using Student’s *t*-test. The optimal cutoff values of PM average values were determined using Youden’s index. Binomial logistic regression was performed to assess the independent predictive value for PM.

## 3. Results

### 3.1. Participant Characteristics

Of the 79 participants diagnosed with COPD included in the study, 39 were classified as reporting frequent exacerbations. The average age of the included participants was 65.49 years, most of them being men, and residing in urban areas. There was no significant difference between participants with frequent exacerbations compared to those with infrequent exacerbations. In addition, no statistically significant difference was found between the two groups according to the type of cooking source used. However, there is a significant difference according to the type of energy used to heat the house (*p* = 0.01) (Table 1).

### 3.2. Relationship between Air Quality and COPD Frequent Exacerbation

**Figure 2:**
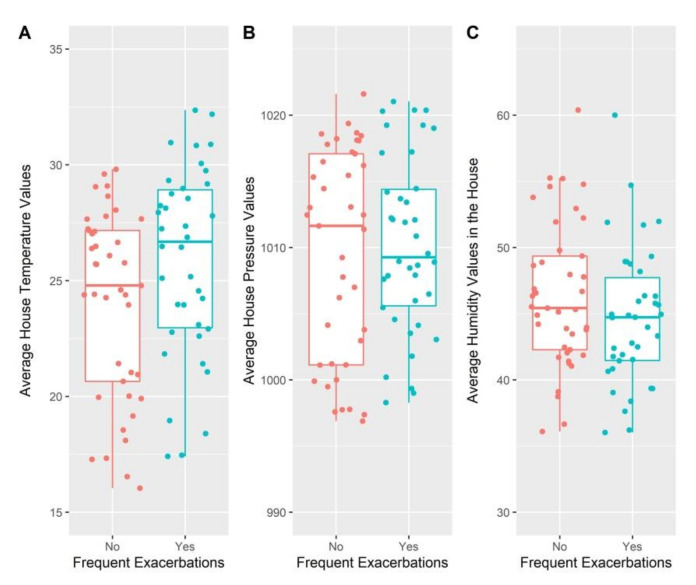
Comparison of the average values of temperature (**A**), atmospheric pressure (**B**) and humidity (**C**) in the participants’ residences according to the exacerbation frequency.

Average temperature values in the houses of patients with frequent exacerbations were higher than those in the houses of patients with infrequent exacerbations (24.78 °C vs. 26.67 °C) (Figure 2). However, this difference was not statistically significant (*p* = 0.43). The average values of atmospheric pressure and humidity were lower in the houses of patients with frequent exacerbations (1009.25 vs. 1011.64, respectively, 44.72 vs. 45.42); however, these differences were also not statistically significant.

In addition, when comparing the minimum and maximum values of temperature, humidity, and atmospheric pressure in the participants’ houses, no statistically significant difference was identified between the two groups (infrequent exacerbations vs. frequent exacerbations) (Table 2).

**Figure 3:**
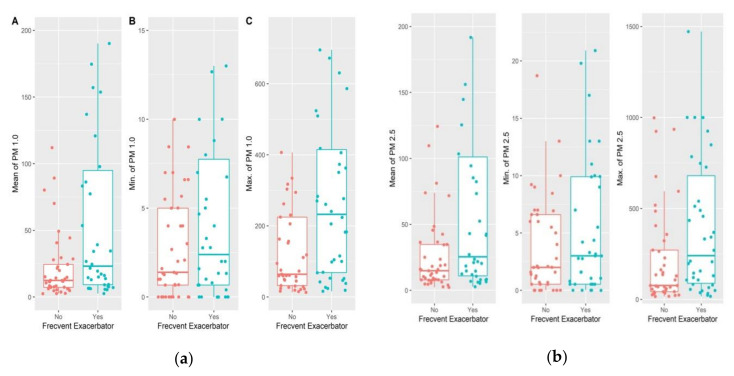
Difference in PM values at participants’ houses according to frequency of exacerbations: (**a**) Average overall (A), minimum (B) and maximum (C) PM 1.0 values; (**b**) Average overall, minimum and maximum PM 2.5 values.

**Figure 4:**
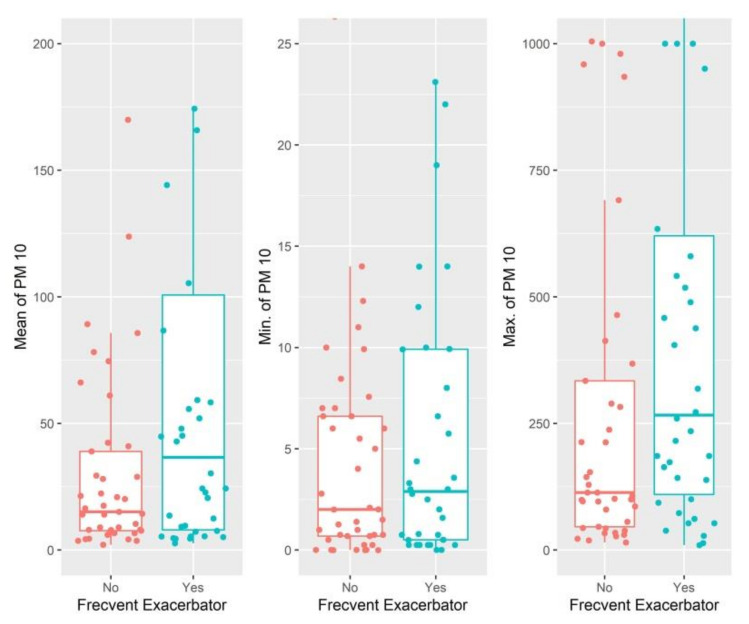
Difference in average PM 10 values during monitoring at participants’ homes according to frequency of exacerbations.

The average PM 1.0, PM 2.5 and PM 10 values during the study period had a median of 12.35 (17.28), 14.83 (26.79), and 15.05 (31.24), respectively, in the group of participants with infrequent exacerbations, and 23.04 (85.76), 25.32 (90.17), and 36.58 (92.77), respectively, in the group with frequent exacerbations (Figure 3 and Figure 4). However, the difference was found to be significant only for PM 1.0 values.

Logistic regression was used to analyze the relationship between PM values and the likelihood of experiencing frequent exacerbations. The results, presented in Table 3, show that for every one unit (1 µg/m^3^) increase in average PM 1.0, PM 2.5 and PM 10, the risk of frequent exacerbations increases by 1.7%, 1.4% and 1.0%, respectively.

**Figure 5:**
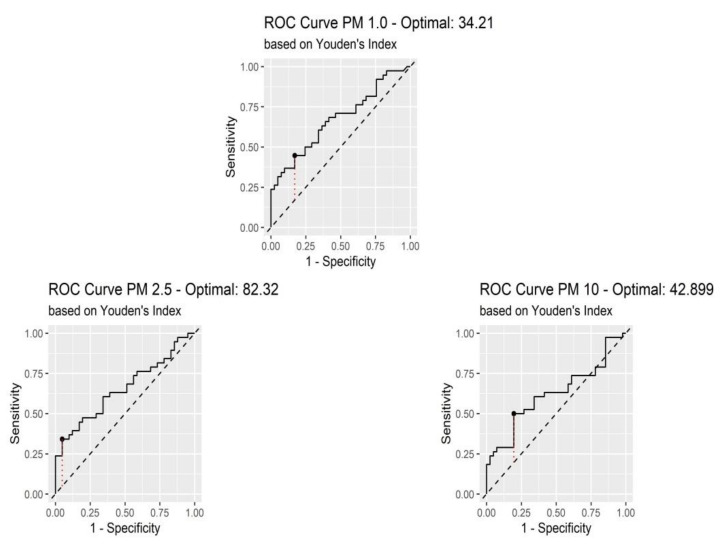
Receiver operating characteristic (ROC) curves of PM in predicting frequent exacerbations.

Receiver operating characteristic (ROC) curves of average PM values were created to determine whether their baseline was predictive of frequent exacerbations in COPD patients (Figure 5). In addition, the Youden index was used to determine the optimal cut-off values.

The areas under the curves (AUCs) of PM 1.0, PM 2.5 and PM 10 were 0.673, 0.654, and 0.622, respectively. The optimal cutoff values obtained from Youden’s index are shown in Table 4.

A univariate regression analysis was conducted to determine the relationship between average PM values (below or above cutoff value) and frequent exacerbations in COPD patients. Patients with average PM 1.0, PM 2.5 and PM 10 values in their homes are 3.93, 10.14 and 4.12 times more likely to suffer frequent exacerbations, respectively (Table 5).

Furthermore, when adjusting odds ratios for age, gender, smoking status, number of packs of cigarettes smoked per year, and comorbidities, the results show that patients with PM 1.0, PM 2.5, and PM 10 in their homes above the cutoff values, are 5.16, 31.03, and 12.99 times more likely to experience frequent exacerbations, respectively. However, statistically significant results were observed only for PM 2.5 and PM 10 (Table 6).

## 4. Discussion

The aim of this study was to assess the degree of microparticulate pollution in the houses of COPD patients and to determine whether the values recorded are correlated to COPD exacerbations.

Sources of particulate matter in the home can be extremely varied: smoking, cooking, heating, passive smoking, use of cleaning products, and even inhaled pollutants from outside. Patients with severe and very severe forms of COPD spend more time indoors because they are unable to leave the house due to the fact that most of them have symptoms or are oxygen-dependent. Thus, it is very important to measure indoor air quality more accurately for these patients than those measured using larger stations located in cities [2,10,11].

In our study, we observed that PM 1.0, PM 2.5 and PM 10 values recorded in participants with frequent exacerbations were higher than in residents of patients with rare exacerbations. However, these differences were statistically significant only for PM 1.0. In a study by Osman et al. on COPD patients, the values recorded were four times higher than the guidelines defined by the US Environmental Protection Agency [12].

Another similar study was conducted in Taiwan, but it was conducted on a group of 19 patients with COPD and a wide variety of lung damage, from early-stage to very severe [7]. Our study focused on severe and very severe forms of the disease, with patients having similar spirometric characteristics; thus, the impact is more specific.

Although the risk factors for exacerbations are diverse, air pollution is an independent and important one in COPD exacerbations. Short-term exposure to various air pollutants has been associated with an increased risk of AE [13,14]. In a study conducted on 4761 patients, we see that each 10 µg/m^3^ increase in PM 2.5 concentration on the concomitant day of onset of an acute exacerbation was associated with a 1.05% increase in developing it [15]. Similar studies showed that for each 10 µg/m^3^ increase in PM 2.5, there is a variable risk in developing an exacerbation [16,17]. After all, we observed in our study that for every 1 µg/m^3^ increase in average PM 2.5 values, there is a 1.8% increase in the risk of developing exacerbations. In addition, a 1 µg/m^3^ increase in mean PM 1.0 and PM 10 increased the risk of exacerbation by 1.7% and 1%, respectively. Moreover, an average value of PM 1.0, PM 2.5 and PM 10 above 32.21 µg/m^3^, 82.32 µg/m^3^, and 42.89 µg/m^3^ increases the likelihood of an exacerbation by 3.83, 10.14, and 4.12 times, respectively. Morantes-Caballero et al. showed that patients with acute exacerbation of COPD had a higher median of particulate matter 48 h before the onset of symptoms [18]. In addition, Liang et al. showed that the most susceptible patients to air pollutants were women and subjects over 65 years of age [19].

Furthermore, in a multicenter study conducted in four different countries on 135 patients, no correlation was observed between air pollution levels and lung function. However, this parameter is subjective because the patients monitored in our study were in severe and very severe stages using daily inhalation medication [20]. In contrast, Gao et al. observed that short-term exposure to PM 2.5 may decrease FVC percentage in COPD patients [21]. Furthermore, in a study conducted in Thailand, FEV1 and FVC respiratory functional parameters were significantly lower when PM 10 levels increased during a seasonal smog period [22]. Lee et al. showed that PM 1.0 and PM 2.5 are closely related, their compounds containing carbonaceous aerosols in the composition: 45.7% and 44.4%, respectively, showing that PM 1.0 is a better indicator for gases emitted by vehicles [23].

Our study has some limitations. First of all, it included only a small group of 79 patients diagnosed with severe and very severe forms of COPD. This proved that the study also required monitoring patients with other COPD phenotypes (chronic bronchitis, emphysema, overlap asthma-COPD (ACO) and overlap bronchiectasis-COPD (BCO)). Secondly, the level of micro-particles, temperature and humidity inside a home varies greatly depending on who lives in it and what activities take place within it. Moreover, this device was not attached to the patient to monitor the PM that they inhale, but rather measured the PM of the entire house. We installed the device in a place where the patients spend most of their time. A longer observation period would strengthen the study. PM was measured over a short period of time; the values within this period could have been different if they had been monitored for a longer period, and it would have been ideal to be able to analyze the composition of PM. In order to measure the degree of pollution that these patients are affected by, a constant measurement should be performed, but most patients do not agree because their personal space is invaded for too long. In addition, a longer observation period would be more relevant. Further studies should focus on monitoring for a longer period of time the inhaled micro-particles at an individual level and analyzing their composition. In addition, the AUC-ROC for all variables is below 0.7. Even though the values are above 0.5, and therefore still able to discriminate between two groups, these values are below the acceptable level of 0.7–0.8.

## 5. Conclusions

Our study showed that patients diagnosed with COPD who have a frequently exacerbating phenotype live in homes where the values of micro-particles PM 1.0, PM 2.5 and PM 10 are significantly increased compared to those of infrequently exacerbating patients. In this study, 79 patients were monitored and, as far as we know, it is the first study of its kind in Romania, with the crucial differentiator being the monitoring and measuring of the microparticles within the patients’ homes and not in the outdoor environment.

In order to manage and improve the quality of life, further studies are required to quantify the effects of air pollution on these types of vulnerable patients.

## Figures and Tables

**Table 1:** Characteristics and living conditions of the 79 participants included in the study.

	Total	Frequent Exacerbation		
		No	Yes	*p* Value
Age	65.49 ± 9.56	67.36 ± 9.21	63.47 ± 9.64	0.07
*Gender*				
Female	18/22.8%	10/24.4%	8/21.1%	0.79
Male	61/77.2%	31/75.6%	30/78.9%	
*Residence*				
Urban	61/77.2%	34/82.9%	27/71.1%	0.28
Rural	18/22.8%	7/17.1%	11/28.9%	
House Surface	59 (43)	64.0 (41.0)	56.5 (37.5)	0.82
*Type of cooking source*				
Gas	75/94.9%	39/95.1%	36/94.7%	0.94
Electric	2/2.5%	1/2.4%	1/2.6%	
Biomass	2/2.5%	1/2.4%	1/2.6%	
*Type of heating*				
Gas	59/74.7%	36/87.8%	23/60.5%	0.01
Electric	4/5.1%	0/0.0%	4/10.5%	
Biomass	16/20.3%	5/12.2%	11/29.8%	
*Smoke status*				
Never	1/1.3%	1/2.4%	0/0.0%	-
Former	50/63.3%	29/70.7%	21/55.3%	0.16
Current	25/31.6%	10/24.4%	15/39.5%	0.22
Secondhand smoke	37/46.8%	14/34.1%	23/60.5%	0.02
Home Oxygen	49/62.0%	22/53.7%	27/71.1%	0.08
*Comorbidities*				
Asthma	11/13.9%	6/14.6%	5/13.2%	0.55
Bronchiectasis	10/12.7%	3/7.3%	7/18.4%	0.12
Tuberculosis	21/26.6%	8/19.5%	13/34.2%	0.20
Hypertension	67/84.8%	38/92.7%	29/76.3%	0.06
Heart diseases	51/64.6%	29/70.7%	22/57.9%	0.25
Diabetes	10/12.7%	7/17.1%	3/7.9%	0.31
*Spirometry*				
FEV1 (%)	33 (15.5)	37.3 (14)	29.0 (13.75)	0.01
FEV1/FVC (%)	51 (14.25)	52.44 (10.49)	45.55 (11.12)	0.006
FEF 25–75%	13 (8.7)	16.7 (8.9)	11.5 (3.0)	<0.001
*COPD Stages*				
GOLD 3	47/59.5%	29/70.7%	18/47.4%	0.04
GOLD 4	32/40.5%	12/29.3%	20/52.6%	

COPD = Chronic obstructive pulmonary disease; FEV1 = Forced expiratory volume in the first second; FVC = Forced vital capacity; FEF25–75% = forced expiratory flow at 25–75% of the pulmonary volume; GOLD = The Global Initiative for Chronic Obstructive Lung Disease.

**Table 2:** Atmospheric parameters in the home settings of 79 COPD patients.

Variable	Total (Median [IQR])	No Exacerbation (Median [IQR])	Frequent Exacerbation (Median [IQR])	*p*-Value
***House Temperature (°C)***				
Minimum values	20.23 (8.21)	18.87 (7.02)	21.96 (7.88)	0.37
Maximum values	28.54 (4.81)	27.92 [(7.65)	28.99 (5.91)	0.31
Average	25.72 (6.37)	24.78 (6.91)	26.67 (5.95)	0.43
***House Humidity***				
Minimum values	39.00 (6.5)	39.00 (6.50)	38.25 (6.83)	0.49
Maximum values	55.37 (9.50)	55.50 (13.0)	55.20 (8.60)	0.82
Average	44.95 (7.01)	45.42 (7.08)	44.72 (6.26)	0.26
***House pressure***				
Minimum values	1004.61 (12.91)	1007.05 (14.62)	1003.99 (11.76)	0.50
Maximum values	1015.6 (9.94)	1015.86 (16.85)	1015.53 (8.20)	0.82
Average	1010.86 (13.13)	1011.64 (15.96)	1009.25 (8.79)	0.50

**Table 3:** Univariate binary logistic regression analysis between average PM values (continuous scale) and frequent exacerbations.

Variables	B	S.E.	*p* Value	OR	95%CI	
					Lower	Upper
PM 1.0 (µg/m^3^)	0.017	0.007	0.01	1.017	1.004	1.030
PM 2.5 (µg/m^3^)	0.014	0.006	0.01	1.014	1.003	1.026
PM 10.0 (µg/m^3^)	0.009	0.004	0.03	1.010	1.001	1.018

B = the unstandardized regression weight; CI = confidence interval; S.E. = standard error; OR = Odds Ratio; PM = Ambient particulate matter.

**Table 4:** Area under ROC curves, Youden index, and optimal cutoff.

Variables	AUC	Youden	Optimal Cut-Off	Sensitivity	Sensibility
PM 1.0	0.673	0.276	34.21 µg/m^3^	44%	82%
PM 2.5	0.654	0.293	82.32 µg/m^3^	34%	95%
PM 10.0	0.622	0.304	42.89 µg/m^3^	50%	80%

AUC = Area under the ROC Curve; ROC = Receiver operating characteristic; PM = Ambient particulate matter.

**Table 5:** Univariate logistic regression analysis of PM values.

Variables	B	S.E.	*p* Value	OR	95%CI	
					Lower	Upper
PM 1.0 > 32.21 µg/m^3^	1.36	0.528	0.010	3.93	1.39	11.06
PM 2.5 > 82.32 µg/m^3^	2.31	0.802	0.004	10.14	2.10	48.79
PM 10 > 42.89 µg/m^3^	1.41	0.510	0.006	4.12	1.51	11.21

B = the unstandardized regression weight; CI = confidence interval; S.E. = standard error; OR = Odds Ratio; PM = Ambient particulate matter.

**Table 6:** Multivariate logistic regression analysis of PM values.

Variables	B	S.E.	*p* Value	aOR *	95%CI	
					Lower	Upper
PM 1.0 > 32.21 µg/m^3^	1.64	0.904	0.069	5.16	0.87	30.38
PM 2.5 > 82.32 µg/m^3^	3.43	1.384	0.013	31.03	2.05	468.09
PM 10 > 42.89 µg/m^3^	2.56	0.970	0.008	12.99	1.94	87.05

* aOR = adjusted Odds Ratio for age, gender, smoking status, packs of cigarettes smoked per year, and comorbidities; B = the unstandardized regression weight; CI = confidence interval; S.E. = standard error; PM = Ambient particulate matter.

## Data Availability

The datasets used and/or analyzed during the present study are available from the first author on reasonable request.
